# Use of phthalocyanine-derived mouthwash as a protective factor for COVID-19: a community trial

**DOI:** 10.3205/dgkh000460

**Published:** 2024-02-21

**Authors:** Verônica Caroline Brito Reia, Fabiano Vieira Vilhena, Heitor Marques Honório, Lucas Marques da Costa Alves, Roosevelt da Silva Bastos, Paulo Sérgio da Silva Santos

**Affiliations:** 1 Department of Surgery, Stomatology, Pathology, and Radiology, Bauru School of Dentistry, University of São Paulo, Bauru, Brazil; 2 Oral Health & Technologies, Bauru, Brazil; 3 Department of Pediatric Dentistry, Orthodontics and Public Health, Bauru School of Dentistry, University of São Paulo, Bauru, Brazil; 4 Hospital Estadual de Bauru, Bauru, Brazil

**Keywords:** COVID-19, antiviral mouthwash, phthalocyanine derivative, protective effect, population study

## Abstract

**Aim:**

In a population profile corrected for sociodemographic factors, the aim of this study was to examine sociodemographic the protective effect of a phthalocyanine-derived mouthwash (APD) before infection with SARS-CoV-2, in addition to analyzing the survival of the at-risk population and the confirmed diagnosis of COVID-19.

**Methods:**

For individuals from the Uru municipality, a structured questionnaire consisting of two parts was completed before the distribution of APD. Subsequently, subjects received two bottles containing 600 mL of APD and were instructed to rinse/gargle with 3 mL of the solution 3 to 5 times per day for 1 min for 2 months. Data were obtained from the electronic system of the municipal health center, organized in a spreadsheet, and analyzed using multiple linear regression and Cox regression analysis.

**Results:**

The study included 995 participants with the following sociodemographic data: 98/995 individuals (p<0.002) who did not complete high school used the APD 66.30 times more than did individuals with higher education. The results in terms of survival were meaningful in relation to the duration of APD use. The protective factor for COVID-19 was 14.1%.

**Conclusion:**

Daily use of a solution containing phthalocyanine derivatives provided a higher protection factor against COVID-19 infection, predominantly in individuals without a school-completion certificate.

## Zusammenfassung

**Zielsetzung:**

: Ziel der Studie war es, bei einem um soziodemografische Faktoren bereinigten Bevölkerungsprofil die Schutzwirkung von Mundwasser auf Phthalocyanin-Basis (APD) vor der Infektion durch SARS-CoV-2 zu überprüfen, zusätzlich zur Analyse des Überlebens der Risikopopulation und in Verbindung mit der bestätigten Diagnose von COVID-19.

**Methode:**

Personen aus der Gemeinde Uru füllten vor der Verteilung von APD einen strukturierten Fragebogen aus, der aus zwei Teilen bestand. Anschließend erhielten die Teilnehmer zwei Flaschen mit 600 ml APD und wurden angewiesen, 2 Monate lang 3 bis 5 Mal täglich 1 min lang mit 3 ml der Lösung zu spülen/zu gurgeln. Die Daten wurden aus dem elektronischen System des städtischen Gesundheitszentrums entnommen, in einem Tabellenkalkulationsprogramm organisiert und mit Hilfe von multiplen linearen Regressionstests und Cox-Regressionsanalysen analysiert.

**Ergebnisse:**

Die Studie umfasste 995 Teilnehmer mit folgenden soziodemografischen Daten: 98/995 Personen (p<0,002) mit einem unvollständigen Schulabschluss nutzten das Mundwasser 66,3 Mal häufiger als Personen mit höherer Bildung; und das Überleben zeigte ein aussagekräftiges Ergebnis in Bezug auf die Zeit der Nutzung der APD-Lösung, der Schutzfaktor für COVID-19 war 14,1%.

**Schlussfolgerung:**

Die tägliche Anwendung einer Mundspüllösung mit Phthalocyanin-Derivaten führte vor allem bei Personen mit niedrigem Bildungsniveau zu einer hohen Schutzwirkung gegen eine SARS-CoV-2 Infektion.

## Introduction

The Severe Acute Respiratory Syndrome Virus-2 (SARS-CoV-2) is the causative agent of the coronavirus 2019 disease (COVID-19), whose rapid global spread is responsible for the pandemic of the same name [1]. Brazil is one of the countries most affected by the pandemic, where more than 12,227,179 cases have been confirmed [1], [2]. The Unified Health System (SUS), the agency responsible for addressing public health threats and managing public health emergencies, was unable to stop the pandemic in Brazil [3].

This phenomenon, combined with the profound socioeconomic inequalities and demographic characteristics of the population, directly affects responses to the pandemic, which may vary depending on the strategic plans implemented by the local government and the response of Brazilian municipalities [4]. However, Brazilian governments have taken successful measures to mitigate the infection and spread of COVID-19, such as by increased focus on hygiene, use of masks, social distancing, as well as the use of antivirally effective antiseptic solutions for rinsing/gargling [5], [6], [7].

SARS-CoV-2 is vulnerable to oxidation; therefore, oral antiseptics containing oxidizing agents [8], including phthalocyanines [9], [10], are recommended to act directly in the oral cavity. The literature states that their derivatives have oxidizing properties and promote self-activation and continuous production of reactive oxygen in the presence of molecular oxygen [9]. This means that the molecules of this compound can induce oxidative stress in microorganisms, leading to their inactivation and inhibiting the growth of infectious particles by contact with oral antiseptics during washing/gargling [9].

In-vitro studies have shown that the use of oral antiseptics at non-cytotoxic concentrations resulted in viral load inactivation of over 99.9% in the individuals analyzed [8], [10], as well as a reduction in hospital stay (from 7 to 4 days) with concomitant clinical improvement in symptoms [6], [9]. These results raise the possibility of using oral antiseptics containing iron phthalocyanine derivatives as an adjunctive tool against COVID-19 for the world population, for which there are no population studies demonstrating the efficacy of the derivative. Thus, the present quasi-experimental, prospective, longitudinal community trial aimed to examine the population profile adjusted for sociodemographic factors and the effect after a certain duration of phthalocyanine derivative-containing mouthwash use on confirmed diagnoses of COVID-19. In additon, this study analyzed the survival rate of the at-risk population and the confirmed diagnosis of COVID-19 starting with the first week of mouthwash distribution.

## Materials and methods

### Characterization of the study and ethical parameters

This study was conducted in the Bauru mesoregion, in which the urban population living in Uru was invited to participate in a quasi-experimental, prospective, longitudinal community study from November 24, 2020, to April 30, 2021, with the highest incidence of COVID-19 (>50 cases/1,000 inhabitants) at the beginning of the study. In addition, the support offered by the municipal health authorities was examined/documented.

The study followed the principles of the Declaration of Helsinki’s ethical standards for human experimentation, as well as biosafety protocols, and was approved by the Human Research Ethics Committee (CAAE 39327120.3.0000.5417). The study was also registered in the Brazilian Registry of Clinical Trials (RBR-6c9xnw3). After the participants were informed about the study and gave their consent, an informed consent form s – “Terms of Consent (TALE)” – and a statement of responsibility from the parents and/or guardians were duly signed after the study had been explained to them and the individuals agreed to participate.

### Characteristics of the reference population

The population of Uru consisted of 1,251 residents according to the last census of 2010, with the number expected to decrease to 1,153 by 2021. 70–85% of them live in urban areas with a demographic density of 8.51 inhabitants/km^2^, a Municipal Human Development Index of 0.712, and a labor force proportion of 15–30%. The Basic Education Development Index concerning public schools in 2019 for individuals in the early years of elementary school was 6.6 and for the final years was 5.6 on a scale of 0 to 10. Further, the municipality has only one access road, one local public health center, two elementary schools and one high school [11].

### Population recruitment and biosafety protocols

The eligibility criteria for inclusion in the study were being a resident of urban and rural areas within 5 km of the city and older than 10 years. Residents who indicated contraindications to oral antiseptic use for medical reasons or who could not gargle or expectorate were excluded.

The principal investigator and her research assistant, who had standard uniform training and acceptable qualifications for conducting the practical portion of the study, visited each household of the Uru municipality to explain the research and provide guidance on the use of mouthwash/rinse with APD. Both examiners followed the biosafety criteria established by the Centers for Disease Control and Prevention (CDC), with the use of an N95 mask, the provision of 70% alcohol, disposable latex gloves, and the maintenance of a distance of 2 meters between the researcher and her assistant and the subjects [12]. No mask was offered as personal protective equipment to the subjects participating in the study.

### Mouthwash intervention protocol

Before the distribution of the APD and the start of the study, each participant completed a structured, 2-part questionnaire developed by the researcher in charge: (I) sociodemographic data and (II) questions specific to COVID-19.

Sociodemographic data included age, gender, educational level, and occupation. The educational level of individuals was divided into 0=no education; 1=did not complete elementary school; 2=completed elementary school; 3=did not complete high school; 4=completed high school; and 5=college level. Regarding occupations, the parameters for classification were taken from the British National Statistics Socio-Economic Classification (NS-SEC) in the three-class version:

1. higher management, administrative, and professional occupations;
2. intermediate occupations, and
3. routine and manual occupations [13].

The specific questions about COVID-19 were as follows: confirmed diagnosis of COVID-19 (yes or no); contact with an infected person (yes or no); if yes, which test was performed for detection (reverse transcription-polymerase chain reaction [RT-PCR], rapid antigen test [RT] or RT-PCR and RT); symptoms (yes or no); if yes, how severe were the symptoms (mild, moderate, severe, critical).

After reading and understanding the risks and objectives of the study and signing the informed consent form, subjects received two 600 mL vials of APD. They were instructed to use 3 mL of the solution alternately as a mouthrinse or for gargling for 1 min, 3 to 5 times a day. The instructions were given in writing, with appropriate information for the age groups of adults and children older than 10 years, and the researcher personally explained the use of the mouthwash to them for two months. During the use of the mouthwash, the team was available to the population to answer any questions about the use of the product.

### Data sources and follow-up

The study used official data from the electronic system of the urban health center for the follow-up period of the participants. These data were entered into the electronic system daily by the urban nursing staff. The data collected were as follows: Date of collection of the COVID-19 test to determine whether the individual was still using the APD at that time, the week he/she received the mouthwash, and, in the case of a positive diagnosis, the time it took to become positive for COVID-19.

Oral antiseptics were distributed for 8 (eight) weeks, and the counting of the follow-up time began at 15, 30, 45, 60, 75, 90, 105, and 120 days (4 months) for the group of subjects who received oral antiseptics in the first week, using the same method in the following weeks of distribution, until the last day of follow-up for the group that received antiseptics in the eighth week.

### Statistical analysis

Data were tabulated and organized in a Microsoft Office Excel 2016 spreadsheet (Microsoft Corporation, Redmond, WA, USA). Descriptive analysis of sociodemographic data, questions specific to COVID-19, and groups of individuals per respective week of oral antiseptic distribution was performed.

In order to determine the independent variables, i.e., which sociodemographic factors were associated with individuals of the study population becoming COVID-19 positive from the start of APD use, a multiple linear regression test was performed using the Jamovi Project 2020 (version 1.2) and R Core Team 2019 (version 3.6) software. The significance level was set at 5% (p<0.05).

To verify survival of the at-risk population and those with a confirmed diagnosis of COVID-19, Cox regression analysis was performed using a hierarchical Wald backward model in IBM SPSS software (version 2.1, IBM; Armonk, New York, USA). Each outcome (individuals at risk and individuals with a confirmed diagnosis) was assessed in the multivariate analysis in five steps. The assumption of proportional hazards for the Cox models was investigated and graphically confirmed by survival functions over time in days using APD. The confidence interval was 95% (95% CI) (significance accepted at p≤0.05).

## Results

**Figure 1:**
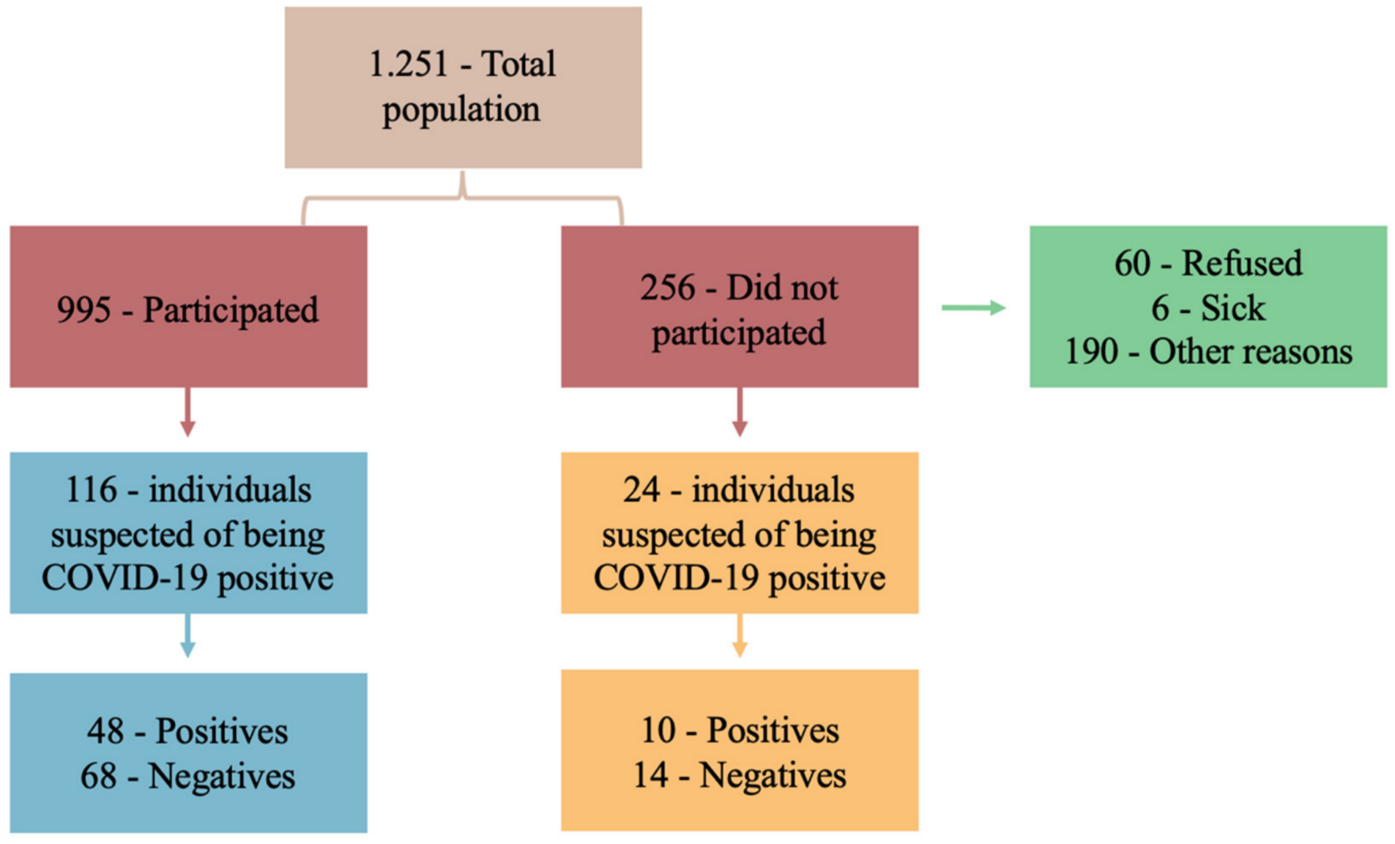
Flowchart of the study sample

Figure 1 (Fig. 1) illustrates the total number of inhabitants living in Uru according to the 2010 census [11], the number of individuals who participated in the study, the respective suspects after the beginning of the study, and the results of the tests to confirm COVID-19 (positive or negative). This was also true for individuals who did not participate in the study. (Tab. 1)

Of the 995 (79.5%) individuals who agreed to participate in the study, 116/995 (11.7%) were suspected of being infected with COVID-19 or at risk thereof after APD use, of whom 48/116 (41.4%) had a confirmed diagnosis of COVID-19 and 68/116 (58.6%) tested negative for COVID-19. Table 1 (Tab. 1) and Table 2 (Tab. 2) show the sociodemographic data of the individuals who agreed to participate in the study and the specific questions related to COVID-19 before the start of the study and before the use of APD. 

Of the 995 (79.5%) individuals, 512 (51%) were female and 483 (49%) were male. The most common age group represented in the study was people 31–45 years of age 266 (27%), followed by the group 46–60 years of age 240 (24%). The predominant level of education was incomplete primary education, held by 389 (39%) individuals, and routine and manual occupations were cited by 832 (84%) municipality residents.

**Figure 2:**
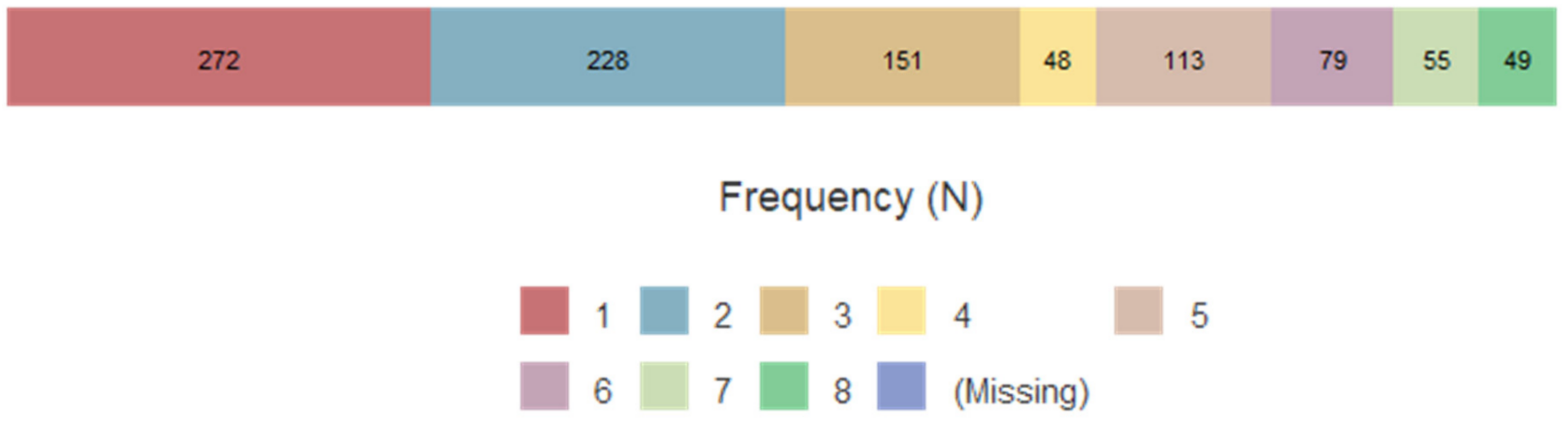
Distribution of oral antiseptics per week

Figure 2 (Fig. 2) illustrates the number of individuals per week of APD distribution. In week 1, 272 (27%) individuals received oral antiseptics; in week 2, 228 did so (23%); in week 3, 151 (15%); in week 4, 48 (4%); in week 5, 113 (12%); in week 6, 79 (8%); in week 7, 55 (6%); and in week 8, 49 (5%) individuals received oral antiseptics. 

Regarding sociodemographic data, individuals with incomplete high school education were estimated to be 66.3 times more likely to use oral antiseptics until they became positive for COVID-19 than individuals with higher levels of education (32.16), a statistically significant result of 98/995 (10%) (p<0.002). Incomplete elementary school education also had a significant value of 389/995 (39%) (p=0.003), with those who used mouthwash 57.78 times more than those with higher levels of education (32.16), with an index of determination (R2) of 0.413. The other sociodemographic data were presented as p=NS values (Table 3 (Tab. 3)).

The survival of these individuals from the first week of distribution of the mouthwash, evaluated in relation to the independent variables, showed a significant value in relation to the duration of use of the solution (Table 4 (Tab. 4)). Thus, the protection factor for COVID-19 during the period of APD use by the individuals participating in the study was 14.1% [100%x0.859 (Coef. B of step 5)=85.9]; (100%–85.9=14.1%] with p<0.05).

**Figure 3:**
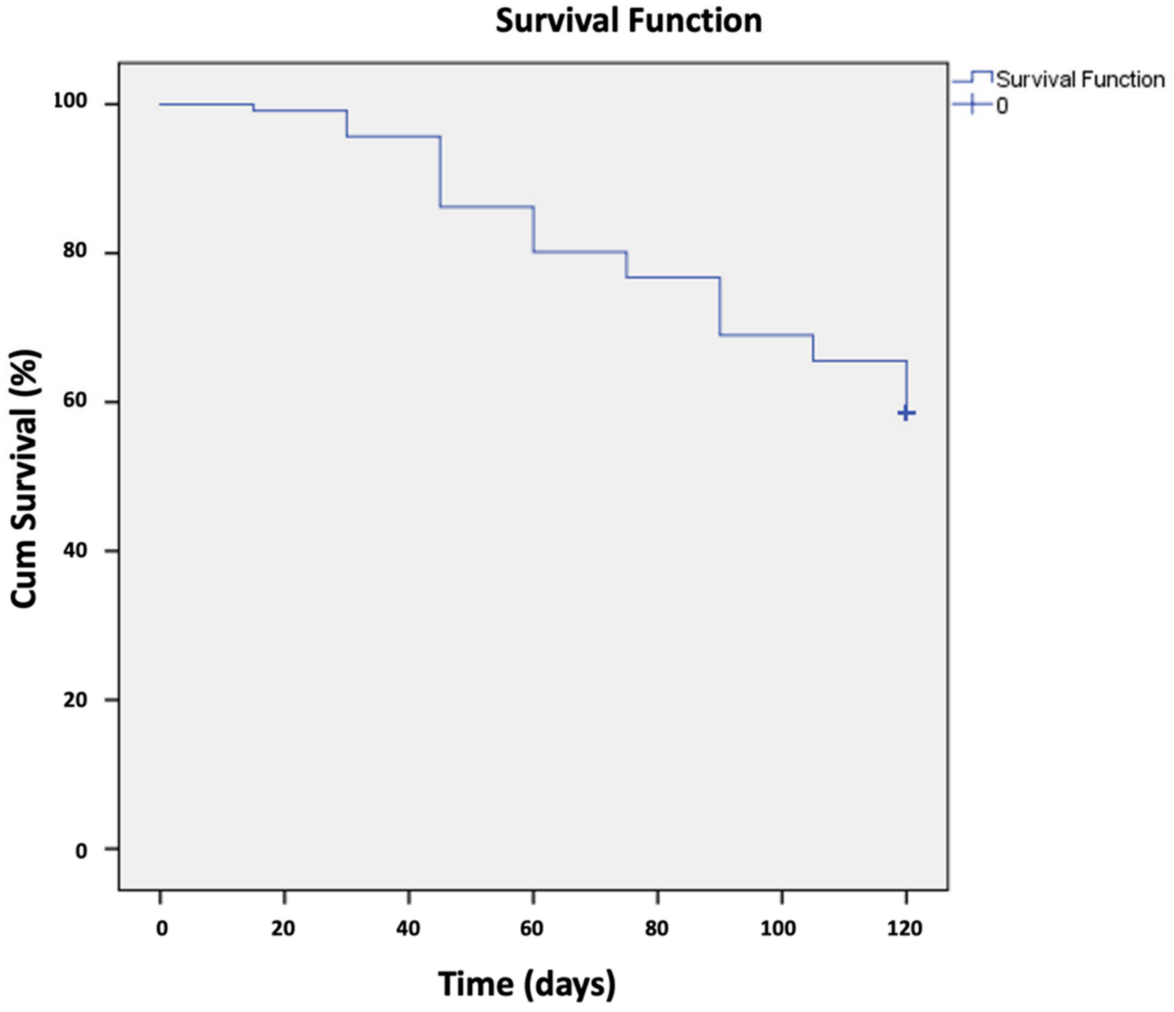
Survival of cases confirmed for COVID-19 by the time of use in days of oral antiseptics.

Figure 3 (Fig. 3) illustrates the survival rate according to the time of application of oral antiseptics 15, 30, 45, 60, 75, 90, 105, and 120 days after the beginning of the study. At day 0, subject survival was 100%, i.e., no positive cases for COVID-19, and remained constant throughout the first 15 days of oral antiseptic use. When the APD solution was used, the protection factor reached 65% at 120 days, the last day of follow-up for the group of individuals who were in the eighth week of distribution. 

## Discussion

Daily use of the APD-containing solution provided a 14.1% protective factor for COVID-19 infection, with better adherence among people with high school and incomplete elementary school education. As no studies for this epidemiological profile were found in the literature for comparison, this can be considered a promising result, as this quasi-experimental community study was based on primary prevention measures [14]. Other studies have shown that the use of APD in non-cytotoxic concentrations can reduce the viral load of SARS-CoV-2 in saliva [8] and serve as a therapeutic adjunct to reduce the severity and risk of COVID-19 transmission [6], [15], [16], [17].

Prior to the distribution of oral antiseptics in the studied municipality, the collected demographic data showed that study participants were predominantly women, corroborating another study, according to which women pay more attention to well-being, health, and quality of life. However, they suffer the most from problems related to anxiety, depression, and stress [18]. Given this scenario, it is worth noting that pandemics rarely affect all ethnicities, genders, and age groups equally. The conditions of and responses to COVID-19 are also unequal, which has been observed in other countries and studies [3], [19]. However, in this study, no statistically significant differences were found in relation to gender for problems related to anxiety, depression, and stress.

In a recent study, it was reported that individuals with lower educational levels have a lower risk of COVID-19 infection than individuals with higher educational levels [20]. This is due to the fact that individuals in the higher education group were least likely to have voluntarily quarantined [21] and received a large amount of information from social media [20]. This study showed that individuals with incomplete high school education (10%) and incomplete primary education (39%) were more likely to use APD until they were positive for COVID-19 compared to individuals with higher education. Thus, it is evident that 49% of the population living in Uru and participating in the study strictly followed the guidelines for using the mouth rinsing/gargle protocol, with APD being the greater protective factor.

The literature shows that RT can be widely used as a mass screening test in the population [22], as it is a feasible and easy-to-perform test that identifies many asymptomatic positive cases and/or cases that are no longer in the active transmission phase. This mass screening was performed every three months in the Uru municipality, and prior to APD distribution, 32% of individuals had contact with another individual who had a positive diagnosis for COVID-19, and 17% of individuals underwent RT. Although it is known that the gold standard and most widely used diagnostic method is real-time PCR (RT-PCR), which detects viral RNA in nasopharyngeal samples, this test requires specialized instruments and trained personnel to perform and is more time consuming [23]. In the community studied, RT helped identify asymptomatic active cases and/or individuals who tested positive for COVID-19 and were no longer in the transmission period. This allowed the health care team to consider the use of preventive methods, such as the use of APD, which was evaluated in this study [5], [6], [7].

Regarding the findings associated with sociodemographic factors and survival of the individuals participating in the study, the duration of APD use was the only significant predictor of prevention and control of infection and transmission of the disease by the individuals. Thus, the chances of a positive diagnosis were lower for COVID-19 in days/use, as some oral antiseptics have viral load-reduction activity against SARS-CoV-2 [6], [24].

When mouthwash was used for two consecutive months, it was observed that the protection factor was 14.1% when used daily for positive cases of COVID-19 [14]. These data suggest that APD was used consistently according to guidelines at the beginning of the study. Even those who intermittently used APD were protected to some extent, since after the 120-day follow-up period, less than half (41.4%) of those at risk had a confirmed diagnosis of COVID-19, with a protective factor of 65%.

This study has some methodological limitations. The team was not able to record the daily use of ADF for each individual or the total number of members living in each household. Therefore, important variables, such as whether COVID-19 infection occurred in members of the same household, or members of other households, could not be collected to control for potential confounders. The presence of a disease that prevented individuals from rinsing or gargling with oral antiseptics may have affected the study as a selection bias. Another important limitation of this study is the lack of a control municipality to compare estimates and results. In any case, at such a volatile phase of the pandemic, it was necessary to maintain social isolation as strictly as possible to minimize the risk of infection during the study.

On the other hand, a strength of this study is that even during the pandemic, oral antiseptics were distributed in a timely manner in compliance with all biosafety protocols [12], and diagnostic data with positive results for COVID-19 were collected through a public electronic system of the municipal health center, allowing long-term comparison with data on the distribution and use of ADF by the municipal population during the study. Finally, the methodology used allowed the analysis and identification of relevant population-level sociodemographic predictors for the development of health protection and prevention strategies against COVID-19. For future studies, a longitudinal design could be considered in which one municipality serves as the control group and another as the case group, using RT-PCR and a placebo test as the gold-standard diagnostic method to compare populations from two or more different cities with the same demographic profile.

## Conclusion

Daily use of the solution containing phthalocyanine derivatives promoted a greater protective factor against COVID-19 contagion, and this condition was more prevalent in individuals with low levels of education.

## Notes

### Funding source

This research was funded primarily by TRIALS – Oral Health & Technologies. Funders contributed to the scope and design of this study; however, they did not influence the collation, management, analysis, and interpretation of the data; preparation, review, or approval of the manuscript; or the decision to submit the manuscript for publication. This study was financed in part by the Coordenação de Aperfeiçoamento de Pessoal de Nível Superior (CAPES), Brazil (Finance Code 001).

### Acknowledgments

We acknowledge TRIALS – Oral Health & Technologies and the Coordenação de Aperfeiçoamento de Pessoal de Nível Superior (CAPES), Brazil, and thank Magali de Brito, the study’s research assistant.

### Authors’ ORCID

- Verônica Caroline Brito Reia:
0000-0003-1352-5474
- Fabiano Vieira Vilhena:
0000-0003-3840-3633
- Heitor Marques Honório:
0000-0003-0231-3409
- Marques da Costa Alves:
0000-0001-9018-6395
- Fabiano Vieira Vilhena:
0000-0003-3840-3633
- Roosevelt da Silva Bastos:
0000-0001-5051-1210
- Paulo Sérgio da Silva Santos:
0000-0002-0674-3759

### Competing interests

The authors declare that they have no competing interests.

## Figures and Tables

**Table 1:**
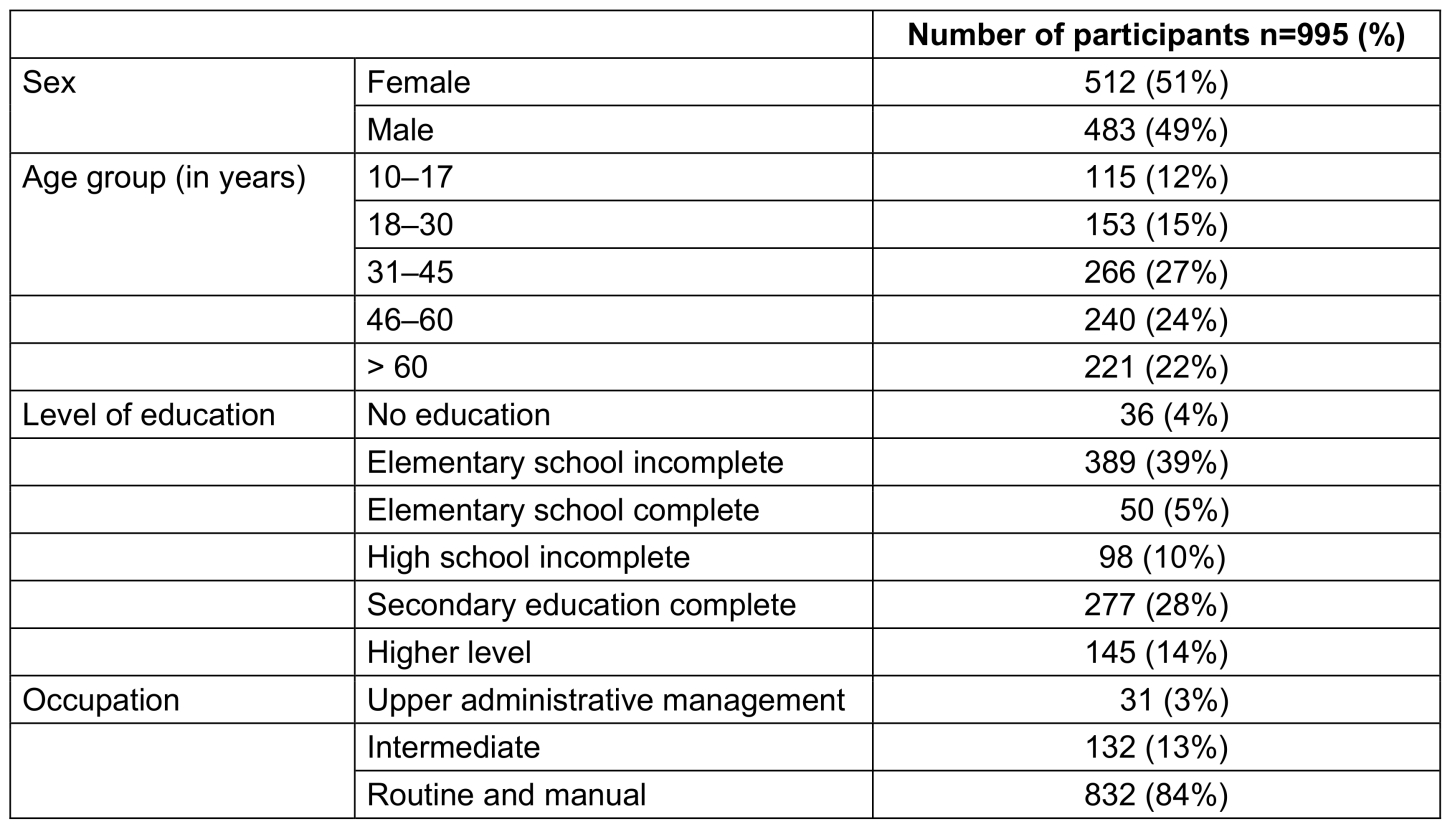
Sociodemographic data of participants in the municipality of Uru in 2020

**Table 2:**
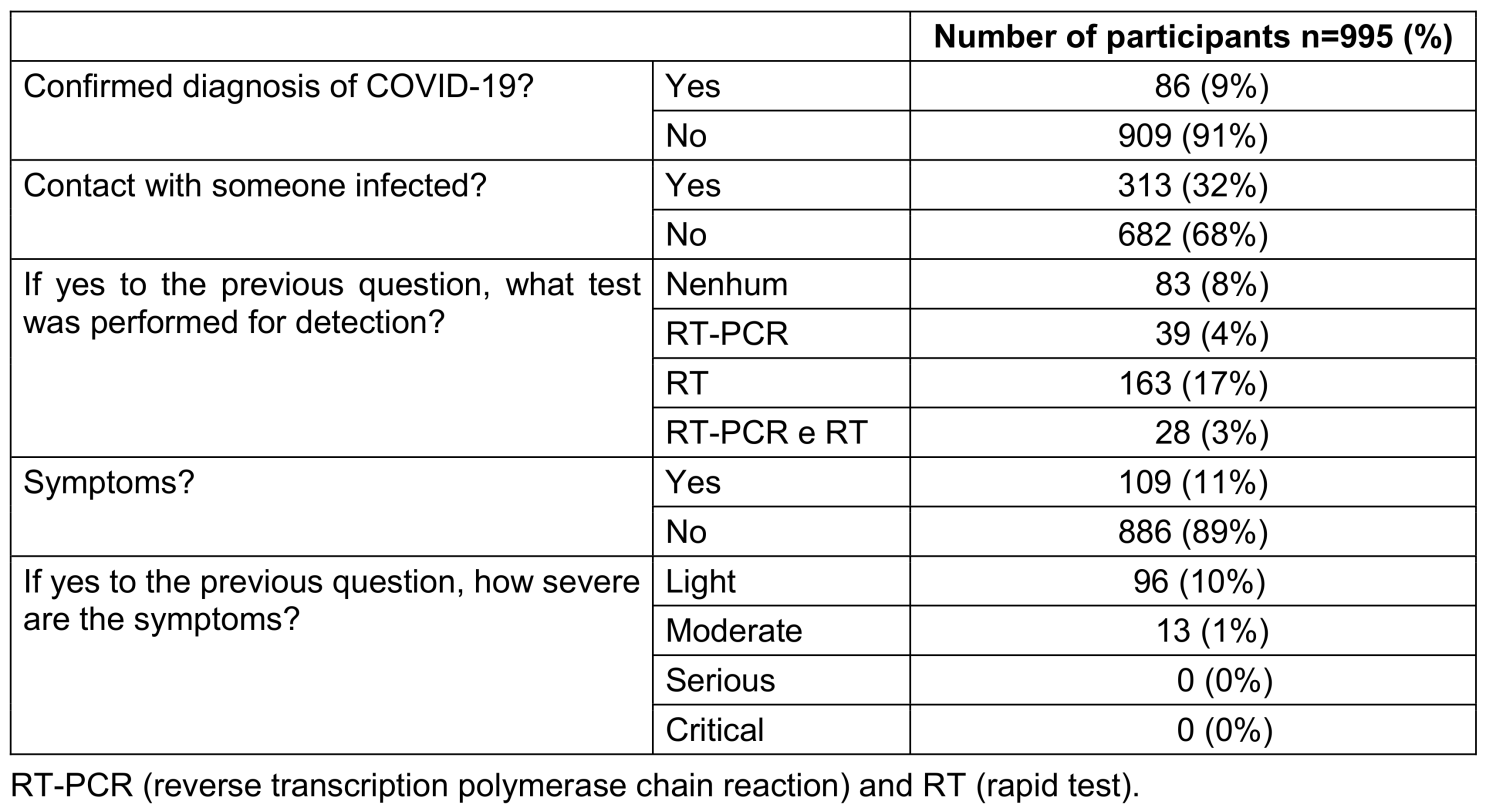
Specific questions related to COVID-19

**Table 3:**
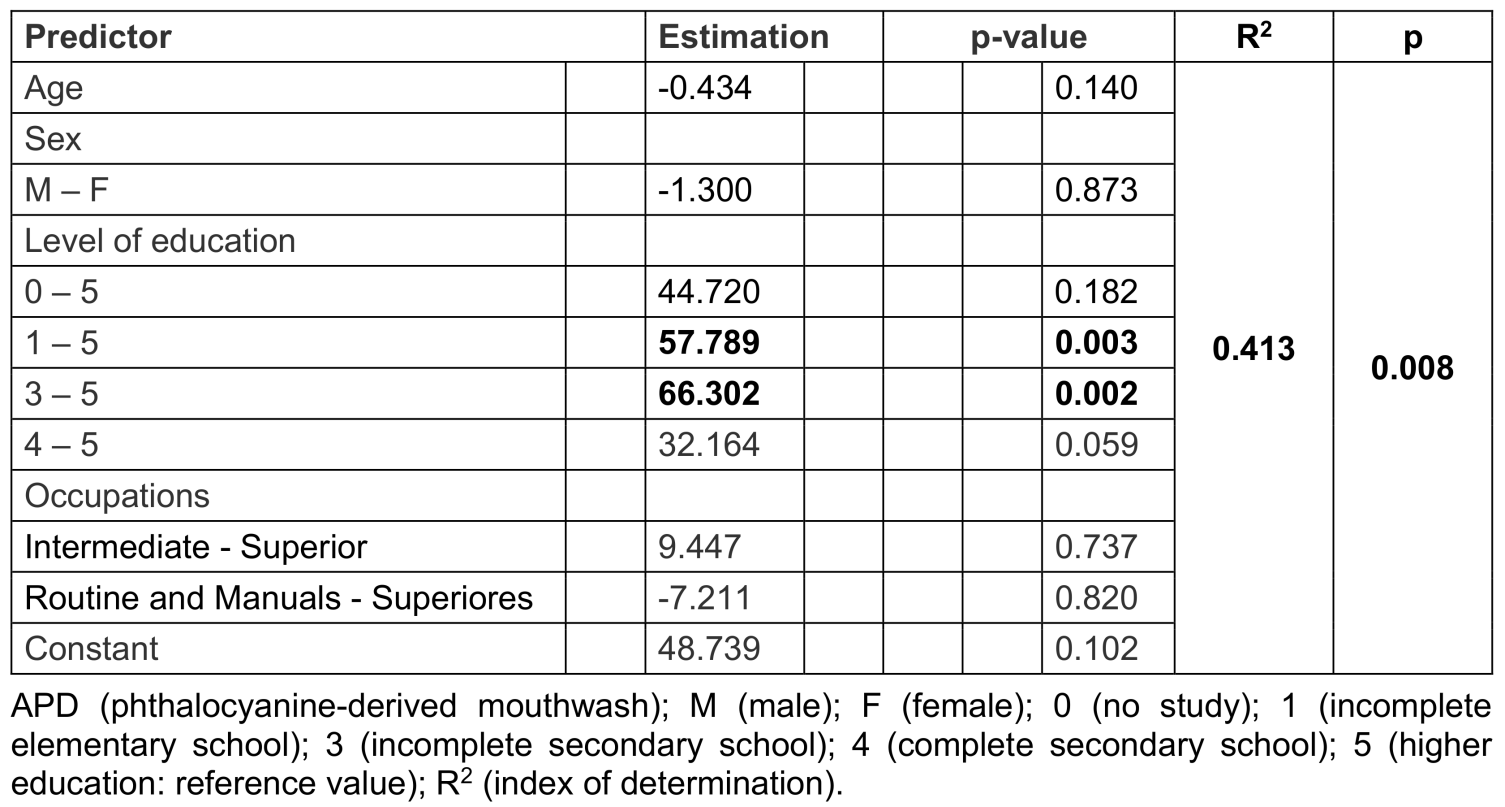
Multiple linear regression between sociodemographic data and the time of use of APD in days

**Table 4:**
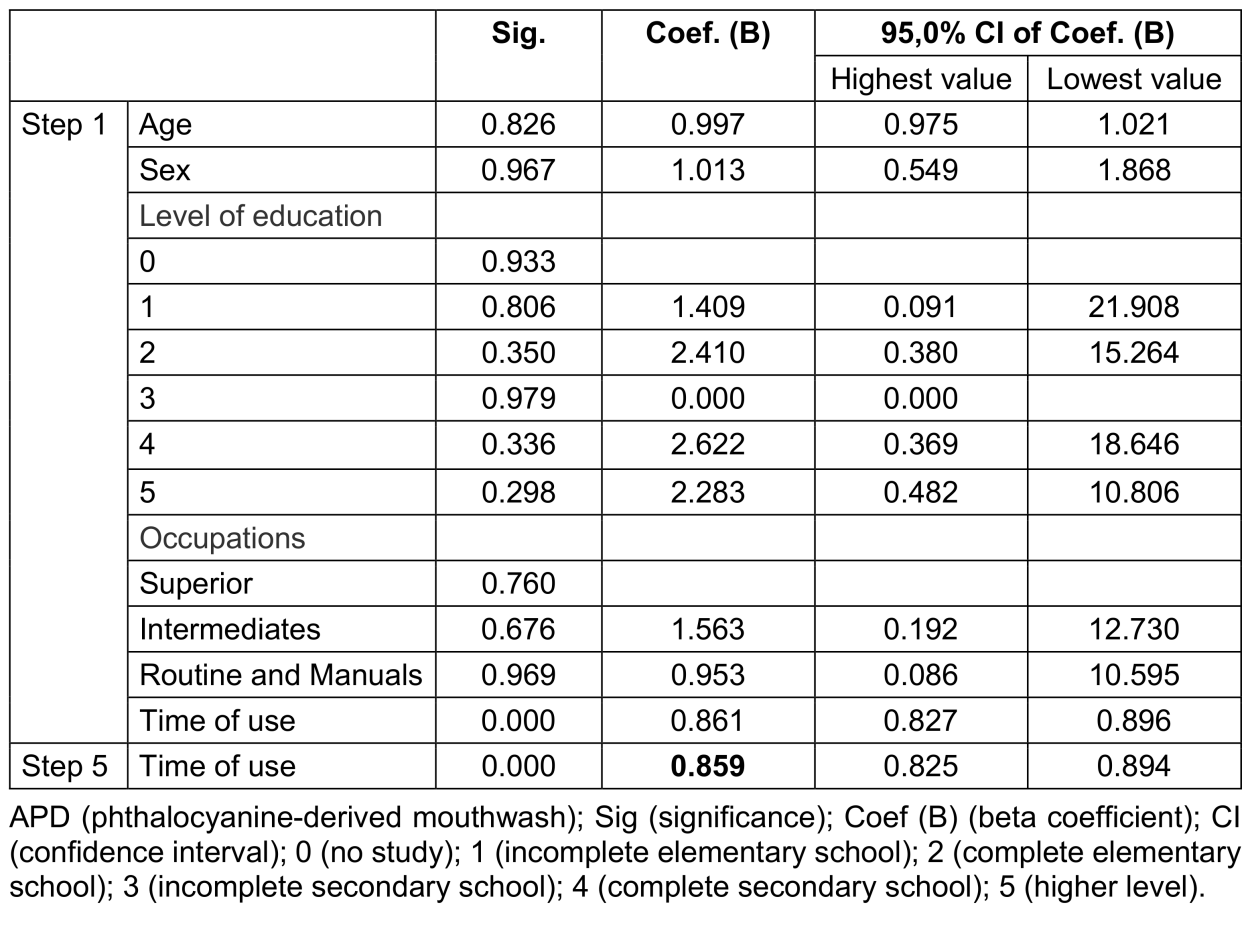
Cox regression between factors associated with independent variables
